# Patients’ and Health Care Providers’ Perceptions on mHealth Use After High-Altitude Climate Therapy for Severe Asthma: Mixed Methods Study

**DOI:** 10.2196/26925

**Published:** 2022-11-22

**Authors:** Rishi Khusial, Sophia van Koppen, Persijn Honkoop, Lucia Rijssenbeek-Nouwens, Karin Berthine Fieten, Sascha Keij, Marieke Drijver-Messelink, Jacob Sont

**Affiliations:** 1 Department of Biomedical Data Sciences, Section Medical Decision Making Leiden University Medical Center Leiden Netherlands; 2 Dutch Asthma Centre Davos Davos Switzerland

**Keywords:** eHealth, mobile health, mHealth, asthma, self-management, home monitoring, mobile phone

## Abstract

**Background:**

Asthma is a common chronic disease with various clinical presentations. Although most patients are able to reach good asthma control, some patients are not able to reach sufficient asthma control following the regular treatment guidelines and could be referred to high-altitude climate therapy (HACT). HACT includes environmental trigger avoidance in the alpine climate with multidisciplinary clinical treatment. Patients with severe and difficult-to-control asthma, who are unable to reach asthma control at sea level, can follow a 12-week lung rehabilitation program at 1600 m above sea level. Mobile health (mHealth) tools can be used to enhance self-management in these patients when they return home. For an mHealth system to be effective, it must meet the expectations of the end users.

**Objective:**

In this Davos@home study, we explored the attitudes toward mHealth aimed at supporting the self-management of patients with severe, difficult-to-control asthma who underwent HACT and asthma health care providers.

**Methods:**

In the first stage, interviews with referrers to HACT and focus groups with patients with asthma who participated in or completed HACT were conducted. The data were then analyzed thematically. On the basis of these results, a questionnaire was developed. In the second stage of the study, this questionnaire, combined with the Asthma Control Questionnaire and the Individual Innovativeness Questionnaire, was provided to patients who completed HACT.

**Results:**

In total, 11 interviews and 3 focus groups (n=18, age 47.6, SD 12.1 years, Asthma Control Questionnaire score 2.6, SD 1.0) were conducted. A total of 3 themes were identified: potential goals, useful measurements, and perceived barriers and facilitators. The questionnaire developed in stage 2 included items based on these results. The most agreed-upon goal among the 52 patients who completed the questionnaire was to increase their asthma control (45/52, 86% of the patients).

**Conclusions:**

Different patients reported that they would benefit the most from different functionalities. Therefore, it is important to tailor functionalities to individual (treatment) goals. When developing an mHealth intervention, it is important to allow personalization to avoid overwhelming the users.

## Introduction

### Background

Asthma is one of the most common chronic diseases worldwide, currently affecting approximately 300 million individuals [1]. Although a majority of the patients can be treated in primary care, some patients have persistent uncontrolled asthma and are referred to specialized asthma care [2]. Within the small group of patients with the most severe, difficult-to-control asthma, standard therapeutic treatments are unable to reach sufficient levels of asthma control [3]. Nonpharmacological interventions, including high-altitude climate therapy (HACT), are proposed for this group of patients [4].

The Davos Dutch Asthma Centre offers patients with severe, difficult-to-control asthma a 12-week high-altitude revalidation program in Switzerland, located 1600 m above sea level [5]. Owing to environmental trigger avoidance in the alpine climate, high-altitude areas may be helpful in to improve asthma control in some patients [6]. A recent study showed improvements in the quality of life and clinical outcomes in patients with severe asthma who completed high-altitude rehabilitation [7]. Following the Dutch guidelines [8], patients are only eligible for HACT when they have uncontrolled asthma (Asthma Control Questionnaire [ACQ] score ≥1.5 [9] and ≥2 exacerbations per year or the use of systemic steroids for ≥6 months per year) despite using high-dose inhalation corticosteroids and long-acting beta-2 agonists [8]. The inhalation technique and medication adherence need to be optimized, and patients are required to have stopped smoking for at least 6 months before referral to HACT is possible. Every year, approximately 80 patients visit the Davos Dutch Asthma Centre.

During their stay in Davos, patients work on different aspects of their rehabilitation, including physiotherapy, asthma education sessions, and self-management skills [10]. On discharge from Davos, the patients are referred back to their pulmonologists in the Netherlands. After discharge, the patients are transferred from 24/7 care to a normal home situation. Between visits to their pulmonologists, patients must manage their asthma themselves. With the help of their health care providers (HCPs), patients engage in self-management [6]. Patients with self-management support after HACT have a lower decline in their asthma control and quality of life compared with patients without self-management support [11]. It is also known that self-management programs have a beneficial effect on asthma control, as reported in a Cochrane review [12]. To improve long-term self-management at home, mobile health (mHealth) systems could be used as self-management support. Using mHealth, the transition to self-management after the patients return from Davos could be softened.

mHealth encompasses the use of mobile devices (such as smartphones, tablets, or wearable devices) to support health care delivery and self-management [13]. Although there are a lot of asthma apps available, not many are clinically validated. During the developmental stage of an mHealth system, it is important to consider the attitudes and expectations of the end users to ascertain the actual use of the system [14]. A user-centered design may allow for the technology to meet the end users’ expectations and, therefore, improve asthma-related outcomes [15]. The effective use of an mHealth tool requires a good fit between the system and end users [16].

### Objective

The first step in designing a user-centered mHealth self-management support system is to determine the attitudes of patients with asthma and their HCPs toward the functionalities the mHealth system should include [17]. This manuscript describes the first part of the Davos@home study, and it is the first step in designing an mHealth system to assist patients with severe asthma with self-management after completing HACT. The aim of this study was to gain insights into the opinions of patients and HCPs on the role of mHealth after HACT to develop an mHealth system that would match their needs and expectations.

## Methods

### Overview

This study consisted of 2 stages. In stage 1, interviews with HCPs (pulmonologists and specialized asthma nurse practitioners) and focus groups with patients with asthma were conducted. Stage 2 involved the development and completion of a questionnaire for patients with asthma who completed HACT in Davos to quantify the opinions generated during the focus groups in a larger population.

### Stage 1

HCPs from hospitals with frequent referrals to HACT in Davos were contacted by the research team for 30-minute interviews in their hospitals. In total, 10 different hospitals with specialized asthma care in the Netherlands were contacted. From every hospital, an interview with 1 pulmonologist and 1 specialized asthma nurse was planned. The interviews were semistructured (Textbox 1 provides a short interview format and the supplement presents the full interview plan). The goal of the interviews was to gain an insight into the attitudes of HCPs toward mHealth-supported self-management. The interviews were conducted between April and June 2019.

Next, a total of 3 focus groups were scheduled. For each focus group, 4 to 8 patients were invited. Patients were eligible if they were aged ≥18 years at the time of the focus group. The inability to understand or speak Dutch was an exclusion criterion. Focus groups were conducted in June 2019.

Every patient who was available in Davos during the visit of the research team was contacted by the medical staff of the revalidation center to participate in the focus groups and was given an information pamphlet. This resulted in 2 focus groups of patients with asthma who were undergoing HACT in Davos and 1 focus group of patients who had completed the HACT program and already returned to the Netherlands.

**Interview and focus group format.**

Interview with health care provider

- Introduction
- Questions relating to
  - Health care providers’ current knowledge and use of mobile health apps for asthma care
  - What measurements would be useful for self-management:
    - Environmental factors
    - Physiological factors
    - Behavioral factors
    - Psychological factors
  - Use of alerts and reminders
  - System-based feedback and recommendations
  - Acceptable time investment
  - Privacy
  - Product design
  - Additional functions
  - Barriers preventing use
- Conclusion and summary

**Focus group with patients**

- Introduction
- Questions relating to
  - Participants’ current knowledge and use of mobile health apps for asthma management
  - What measurements would be useful for self-management:
    - Environmental factors
    - Physiological factors
    - Behavioral factors
    - Psychological factors
  - Use of alerts and reminders
  - System-based feedback and recommendations
  - Acceptable time investment
  - Privacy
  - Product design
  - Additional functions
  - Barriers preventing use
- Participants’ debrief

Upon arrival, the format of the focus group was explained, informed consent forms were signed, and general participant demographics were collected using a short questionnaire. Only the researchers and patients were present during the focus groups. Each focus group discussion lasted approximately 2 hours with a short break in the middle.

The interviews and focus group discussions used a funnel-type method of questioning (from general to specific questions). Before the interviews and focus groups, the researchers constructed an interview plan with questions on different subjects. Prompts were used to probe further details if necessary. The interviews were audio recorded, and the focus groups were video recorded to assist with transcription.

Interviews and focus groups were conducted by RK or SK. RK (BSc in medicine and BSc in political science) is a male medical student and PhD candidate. SK (at the time BSc in medicine) is a female medical student and research intern. The researchers introduced themselves before the interviews and focus groups.

The focus groups and interviews were transcribed verbatim and underwent thematic analysis [18]. RK and SK coded each transcript independently. After transcription, discrepancies between codes or how parts of the transcripts were coded were discussed between RK and SK until consensus was reached [18]. Next, the codes were grouped into different applicable themes. Data management was supported by the ATLAS.ti qualitative analysis software (version 7).

The thematic analyses of the focus groups and interviews resulted in the construction of a closed-ended questionnaire aimed at quantifying the opinions generated during stage 1.

### Stage 2

The web-based questionnaire consisted of 4 parts: baseline patient characteristics, 34 questions based on stage 1 of the study, the ACQ [9], and the Individual Innovativeness Questionnaire (19). The questionnaire generated during stage 1 consisted of questions that participants could rate on a 5-point Likert scale (strongly disagree, disagree, unsure, agree, and strongly agree). Favorable outcomes (agree and strongly agree) were considered as being in agreement with the statement. The Individual Innovativeness Questionnaire is a tool designed to measure individuals’ attitudes toward change, and participants can be fitted into 1 of 5 categories (innovators, early adopters, early majority, late majority, and laggards or traditionalists) [19].

The patient federation *Vereniging Nederland Davos* was actively involved in participant recruitment. They made the complete questionnaire available to patients who they knew had completed HACT in Davos in the past. Patients were also recruited through their social media handles, newsletters, and websites. The questionnaire was administered on the web for 2 months. No formal sample size calculations were performed because statistical analyses were not planned.

### Ethics Approval

This study was conducted in accordance with the principles of the Declaration of Helsinki (2013). The Medical Ethical Committee of the Leiden University Medical Center offered exemption for the ethics approval of the study protocol (P19.039), as it was not required under Dutch law.

## Results

### Participants

In total, 11 HCPs from 7 different hospitals were interviewed, and no replies were received from 3 hospitals. From every hospital, 1 pulmonologist was interviewed, and from 4 of these hospitals, 1 nurse practitioner was also interviewed. In addition, 20 patients with asthma were contacted to participate in the focus groups. Of them, 1 patient declined and 1 patient did not show up at the focus group because of (not study related) sickness. In total, 18 patients with asthma participated in 3 separate focus group sessions (n*=*4, n=6, and n=8 per focus group).

A total of 52 patients completed the questionnaire from stage 2. Table 1 provides a combined overview of the baseline characteristics of the focus group participants and those who completed the questionnaires.

In addition to the described baseline characteristics, we also queried HCPs and focus group patients on the current use of mHealth and eHealth solutions. Overall, 1 HCP used mHealth in relation to immunotherapy, 1 HCP advised a smoking cessation app, 1 HCP used an allergy program (study phase), and 1 HCP had frequent video consultations. Among the focus group patients, 1 patient previously used the Fitbit app, 1 patient used an app named PatientCoach [11] (in the study setting), and 1 patient reported using an asthma diary app.

**Table 1:** Baseline characteristics of the focus group patients and questionnaire respondents.

				Values
**Stage 1 (n=18)**				
	**Sex, n (%)**			
		Female	9 (50)	
		Male	9 (50)	
	Age (years), mean (SD)			49.2 (12.1)
	**Current smoking status, n (%)**			
		Yes	0 (0)	
		No	13 (72)	
		Previously	5 (28)	
	**ACQ^a^ score**			
		Values, mean (SD)	2.1 (1.0)	
		Controlled (<0.75), n (%)	1 (6)	
		Partly controlled (0.75-1.5), n (%)	4 (22)	
		Uncontrolled (>1.5), n (%)	11 (61)	
**Stage 2 (n=52)**				
	**Sex, n (%)**			
		Female	37 (71)	
		Male	15 (29)	
	Age (years), mean (SD)			47.2 (14.5)
	**Current smoking status, n (%)**			
		Yes	0 (0)	
		No	44 (85)	
		Previously	8 (15)	
	Own a smartphone, n (%)			51 (98)
	**Visits to high-altitude climate therapy (times), n (%)**			
		Once	24 (46)	
		Twice	15 (29)	
		≥3 times	13 (25)	
	**ACQ score**			
		Values, mean (SD)	2.6 (1.1)	
		Controlled (<0.75), n (%)	4 (8)	
		Partly controlled (0.75-1.5), n (%)	2 (4)	
		Uncontrolled (>1.5), n (%)	46 (88)	
	**Innovator status, n (%)**			
		Innovators	8 (15)	
		Early adopters	18 (35)	
		Early majority	18 (35)	
		Late majority	7 (13)	
		Laggards, traditionalists	1 (2)	

^a^ACQ: Asthma Control Questionnaire.

### Themes Created in Stage 1

Three distinct themes were identified in stage 1: potential goals, useful measurements, and perceived barriers and facilitators.

#### Potential Goals

In total, 8 main categories of potential goals were formulated based on the data (Textbox 2).

Patients and HCPs reported that mHealth-supported self-management could improve or maintain disease-specific outcomes, including asthma control, quality of life, exacerbation rate, medication adherence, and lifestyle. The HCPs also mentioned that mHealth could reduce the number of in-person consultations by potentially replacing routine meetings with video calls or chats.

Other potential goals of an mHealth system named in the interviews and focus groups were specifically related to HACT. Patients wanted to maintain the (self-management) skills and knowledge they learned during their revalidation and would appreciate to have the education modules from the revalidation available to them electronically so that they could further train themselves.

Furthermore, patients reported that mHealth could improve their disease awareness with the help of monitoring. Being confronted by ACQ data helps the patients understand the severity of their symptoms and, therefore, could help patients respond to their symptoms sooner. Patients feel that they are so used to being sick that they claim to underestimate the worsening of symptoms, and they believe that monitoring could assist their HCPs in better understanding their disease progression. It is important to manage expectations of the end users. Although HCPs reported that they see mHealth primarily as a self-management support tool for patients, the patients would like to see it aid the HCPs in making treatment decisions.

**Potential goals with example quotes.**

**Improving asthma control**

- “That is in fact the most important thing, so the patient should obtain a better quality of life or have a better asthma control.” [HCP3]

**Improving the quality of life**

- “It (the goal) is very simple, the patient should benefit from it [...] having a better quality of life” [HCP3]

**Reducing exacerbation rate**

- “If the app and the patient together have prevented an exacerbation, well yes that would be of course very nice.” [HCP7]

**Improving medication adherence**

- “Because that is it eventually, if you talk about what might be the biggest issue with asthma, it is noncompliance with therapy.” [HCP2]

**Sustaining or inducing lifestyle changes**

- “You know what might be a more important goal? eHealth/mHealth could induce lifestyle changes in a patient.” [HCP1]

**Limiting consultations**

- “And perhaps that it is also more convenient for us [HCPs], and for the patient, that it may save outpatient visits.” [HCP1]

**Retaining high-altitude climate therapy–learned skills and education**

- “What is also important [sic], is that you keep remembering what the advises were from Davos that you received when you were send back home.” [Patient 112]

**Increasing disease awareness**

- “If you are confronted with the data and think, we are not doing so well, that you can intervene faster, which in principle prevents you from getting worse.” [Patient 111]

#### Useful Functions

Five different categories of functions an mHealth self-management intervention could contain were identified: “lifestyle,” “education,” “measurement devices,” “psychological functions,” and “other functions” (Textbox 3).

Within the lifestyle category, some patients wanted to record parameters related to their activity and weight. Other apps that could record food and calorie intake were also mentioned as useful tools that could be incorporated. Exercise was also considered a useful aspect to monitor. Pedometers provide more insights into the number of steps that are taken daily, and a function to set individual goals could motivate patients. However, some patients reported that tracking their physical conditions was not desirable for them. A patient also mentioned that tracking sleep quality would provide more insights into his asthma, as nocturnal symptoms are frequently present in patients with severe asthma. It was said that although it might be difficult to initiate lifestyle changes with just an app, it could support maintaining lifestyle changes.

**Useful functions with example quotes.**

**Lifestyle**

- “But for us it [medication reminders] is not necessary, then again, I do not rule out there are people who certainly do need it.” [Patient 111]
- “Since we often get prednisone or dexamethasone and then our weight after that increases, or some people lose weight. That you also can look at it [an app] and that it is motivating for others to lose weight, and to gain weight again.” [Patient 0217]

**Education**

- “What is asthma actually, a piece of background information, but also if you do not take you medication in time, what happens to the lungs. I got booklets for this, it is something small, but still important.” [HCP5]
- “I want to have it [education modules] as optional. If you think that you want it, that you can easily open it.” [Patient 616]

**Measurement devices**

- “Yes, I think it is like you said, measuring is knowing, how are you doing at the moment. I think that is very important.” [Patient 111]
- “With heart rate, that maybe people could insert a maximum heart rate and if you are too close to it you receive a notification. Often you can feel it already, but there are moments you do not realize it.” [Patient 1116]

**Psychological functions**

- “Well if we are talking about an app, quick access for those who need it with the psychologist or with the care coordinator [from HACT] would be practical.” [Patient 212]
- “Look in the end I think someone still needs a direct conversation. At the moment you fill in ‘well I am feeling a little depressed today’ or ‘I am not really feeling well’ if the app then says ‘come on you can do it!’ or ‘look, the weather is nice outside’ or ‘the glass is half full instead of half empty’...I think you do not what to receive those kind of messages.” [HCP3]

**Other functions**

- “Yes, you receive that [asthma action plan], but with me, it just hangs in my kitchen cupboard. If I open the door I see it and think ‘oh yes, that is useful,’ but I would personally find it convenient if I can find it on my phone.” [Patient 212]
- “To return to the asthma action plan, everything is nicely written down on paper and to be very honest, things like that often ends up in a drawer, the drawer is closed and you never use it again.” [Patient 0716]
- “I am missing the changes in weather for example. Pollen season, all indicators that are available that worsen asthma. It remains often a periodical thing, so yes, it would be nice to find it in there [in an app].” [Patient 516]

Patients reported that they wanted to have some form of education in an mHealth intervention. This varied from more general information about asthma to specific education about exacerbations and breathing techniques they learned during HACT. Patients reported that they also wanted information for their family and friends to help them understand the impact of severe asthma on daily life. HCPs reported that they wanted to have links to videos about correct inhaler use as a reminder for patients, such as the website [20], which has video tutorials about the use of inhalers. Patients from the focus groups reported that these videos could be presented in an app but thought that this might not be useful for themselves but rather for other patients. They also thought that medication reminders would be beneficial for these other patients because they reported that they mostly take their medication as prescribed. A 2-fold reminder system was suggested in a focus group: one reminder to take your daily inhalation medication as a push notification on your phone and another one after some time to check if the medication was indeed taken. With a single press, one should be able to dismiss these notifications.

Different devices were mentioned as useful additions to an effective mHealth self-management intervention. Heart rate and spirometry were often mentioned by patients as indicators that could be measured on a regular basis at home, with the addition of small home monitoring devices. Other parameters named in the focus groups were oxygen saturation, breathing frequency, and blood pressure. The HCPs thought that flooding the patients with numbers generated by these kinds of devices without proper medical guidance would not be helpful, as the patients also need to know what these values mean for their specific situation. Therefore, providing patients with data alone could create a false sense of security.

The patients also mentioned that they wanted a medication counter for their inhalers, indicating how many doses are left. Currently, only a few inhaler manufacturers include a counter on their casings. Patients are afraid that they will run out of (rescue) medication, and a counter would give them a feeling of security. They would like to receive a warning when they are running out of medication.

It was frequently mentioned by HCPs that severe asthma also has strong associations with psychological well-being. Severe asthma impacts the quality of life, and patients are provided with psychological consultations during their HACT revalidation if needed. Although both the patients and HCPs agreed that it would be difficult to incorporate adequate psychological support in mHealth, it is something that should not be overlooked when building an mHealth system. Although human interaction is key to psychological support, an mHealth system could provide a screening tool, for example, for anxiety and depression with the help of a standardized questionnaire [21]. Opinions on this varied among patients; some thought it could be useful, whereas others definitely opposed it.

Other functions mentioned were having access to environmental data because many patients with asthma are hypersensitive to low air quality and allergens, including pollen. Patients and HCPs also wanted to monitor asthma-related parameters using questionnaires, including the ACQ and Asthma-Related Quality of Life Questionnaire. The frequency at which these questionnaires were suggested to be filled out varied between weekly and every 6 months or only when increased symptoms are experienced.

It was suggested that an asthma action plan be included in the app so that it would be available to patients when needed. Patients also wanted to have an option to share the data with their care team, including their pulmonologist, specialized asthma nurse practitioner, and, if necessary (varying per patient), general practitioner and physiotherapist. They would like to see treatment decisions based on the data collected, although this is not the primary aim of self-management. HCPs would also like to see the data of their patients if they help them make more informed decisions or gain insights into their patients’ symptoms.

#### Perceived Barriers and Facilitators

As an intervention is only effective if it is actually used, patients and HCPs were asked about the barriers that prevent them from using an mHealth system (Textbox 4 provides example quotes). The most important barrier that the patients perceived was ineffectiveness. If they feel that the mHealth system does not support them in reaching their goals, they do not use it.

**Perceived barriers and facilitators with example quotes.**

**Effectiveness**

- “It should not be a gadget that delivers more work to the patient and us and, like, in outcome in care is useless.” [HCP1]

**Time consumptions**

- “The majority of thing should be automatic, so it will not cost us time, that it will not be a real burden. Look, if you tell me, like, I would think a minute a day is kind of acceptable to be actually actively involved with things.” [Patient 116]

**Too much information**

- “I do not think it should be too full [of option], then it will not be attractive, it would seem to me.” [Patient 0217]

**Too complicated**

- “But I think it will be very difficult to implement if it gets too complicated. So too many extra clicks on buttons, extra application and things.” [HCP 6]

**Privacy**

- “I think I want it to be really secure [privacy protected], because it is really personal data. Because your things [data] from your pulmonologist are in it, from you psychologist are in it, a lot will be in.” [Patient 212]

**User-friendliness**

- “It should be very user-friendly and I think it will be different in the future, that we can even have mHealth consultations.” [HCP6]

**Personalized**

- “I think the action plan is practical, yes. But what would be even more practical is if you are able to adjust it. So if you return home, and things are changed in the Netherlands by your pulmonologist or whomever. So they can adjust it.” [Patient 212]

Another important barrier is the time required for using the system. Patients would not use the system if entering data would take too much time. The use of automated data collection (with, eg, additional wearable devices) would partially address this problem. Patients were willing to wear additional devices, given that they were hypoallergenic, and if visible, it would be preferred if the devices were fashionable. The system must be part of their daily (or weekly) routine.

In addition to time consumption, the system should not contain too much information or be overly complicated. Patients were willing to use the system if the minimally required use to be effective was limited. The number of questionnaires that the patients were willing to fill varied, but they generally agreed that having a daily question to quantify their disease burden was too much work for insufficient disease gain. Implementing all previously named options into a single system could create an overly comprehensive system, which would prevent actual use because not all functions would be useful for all patients.

Privacy is also an important topic to consider, and the data need to be protected, as they are health data. Too many security measurements and log-in screens negatively affect user-friendliness.

#### Quantifying the Patient Opinions (Stage 2)

Stage 2 participants were predominantly female (37/52, 70%), and the vast majority had uncontrolled asthma (46/52, 89%; Table 1). The results of the questionnaire are provided in Table 2. Patients were positive with respect to all potential goals of an mHealth system but were least positive about the possibility of preventing asthma exacerbation (31/52, 60%). With respect to potential use, the strongest agreement (>80%) was with the functionality to contact their care team; have information for important others; have their personal asthma action plan and outdoor air quality information available; and keep track of their rescue medication, lung function, and exercise behavior. However, only 69% (36/52) of the patients were willing to wear an additional wristwatch to track this. If a trade-off needed to be made between privacy and user-friendliness, 56% (29/52) of the patients preferred privacy.

In total, 65% (34/52) of the patients reported that they would like to have a smart inhaler that could record when they used their inhaler, and 58% (30/52) of the patients wanted medication reminders, although 78% (41/52) of the patients reported that they never forgot to take their medication (the remaining 11/52, 22% of the patients reported forgetting medication only sometimes).

In addition, 88% (46/52) of the patients found it important to customize an app according to their needs.

**Table 2:** Distribution of results from the stage 2 questionnaire study.

		Strongly disagree, n (%)	Unsure, n (%)	Strongly agree, n (%)
**Potential goal of an mHealth^a^ system**				
	Enhance the quality of life	4 (8)	10 (19)	38 (73)
	Make me more aware of my asthma	6 (12)	4 (8)	42 (80)
	Improve asthma control	4 (8)	3 (6)	45 (86)
	Prevent an asthma exacerbation	7 (14)	14 (30)	31 (60)
	Bring about a healthy lifestyle	9 (18)	7 (13)	36 (69)
	Retain what I learned in Davos	3 (6)	0 (0)	49 (94)
	Monitor my symptoms	2 (4)	3 (6)	47 (90)
**I would like to**				
	Receive medication reminders	19 (37)	3 (6)	30 (58)
	Digitally track how often I use my maintenance medication	13 (25)	5 (10)	34 (65)
	Digitally track how often I use my rescue medication	5 (10)	4 (8)	43 (83)
	Keep contact with the care team from Davos	2 (4)	7 (13)	43 (83)
	Have routine outpatient visits to be digital rather than in person	18 (35)	8 (15)	26 (50)
	Receive psychological assistance through an app	16 (31)	9 (17)	27 (52)
	Receive tips on inhalation technique through an app	28 (54)	5 (10)	19 (36)
	Receive asthma education through an app	11 (21)	11 (21)	30 (58)
	Have information for family or friend through an app	9 (18)	1 (2)	42 (81)
	Have the asthma action plan available on my smartphone	4 (8)	2 (4)	46 (88)
	See the outdoor air quality on an app	6 (8)	3 (6)	45 (87)
	Monitor my lung function at home with a spirometer	3 (6)	6 (12)	43 (83)
	Keep track of my diet through an app	14 (27)	6 (12)	32 (62)
	Keep track of my daily steps	7 (14)	2 (4)	43 (83)
	Wear a heart rate monitor or pedometer as a wristband on daily basis	8 (16)	8 (15)	36 (69)
	Have a device that monitors my inhalation technique	14 (27)	4 (8)	34 (65)
I think it is useful for my asthma to monitor my heart rate through a wristband		10 (20)	9 (17)	33 (63)
I think it is important that I could customize an app to my needs		1 (2)	5 (10)	46 (88)

^a^mHealth: mobile health.

## Discussion

### Principal Findings

This study showed that patients and HCPs expressed many different potential goals for an mHealth self-management support system and thought that numerous functions were useful to them. At the same time, having an overly comprehensive system would prevent them from using it. Different patients reported that they would benefit the most from different functions, and making all functions available to all patients could overwhelm the patient, possibly limiting the use of the system. Therefore, creating a one-size-fits-all mHealth system is not the best strategy.

### Personalization

As no 2 patients with asthma are the same, it is important to consider having different functions for different patients. In this group of patients with most severe asthma, some patients might benefit from reminders to take medication, whereas other patients might feel that these constant notifications interfere too much with their daily life and, because of these reminders, are more likely to ignore the app altogether. Many HCPs said that particular functions might work for some patients, but not for others, depending on the skills and willingness of the patient.

There is also a discrepancy between the views of HCPs and patients on the use of e-consultations. With e-consultations, patients could send messages to their HCP whenever they had a question regarding the management of their asthma. HCPs thought that new forms of HCP-patient communication could be more convenient for the patient. However, only 50% (26/52) of the patients from stage 2 reported that they wanted to replace their routine visits with e-consultations. HCPs reported that mHealth is unlikely to fully replace regular treatment; however, it could be used as an additional support tool.

Currently, many of the functions mentioned in the interviews and focus groups are already available in different apps or systems that focus on one specific function. However, it seems to be practical to integrate these numerous functionalities into a single system so that the patients do not have to familiarize themselves with all the different systems. Therefore, it is suggested that a single mHealth self-management support system that encompasses all these different functionalities be created. Patients in the focus groups also reported that they have more trust in a system developed and used in a care setting than in a system produced by a profit-based company.

According to international asthma guidelines, a (personalized) asthma action plan should be the backbone of asthma self-management. These guidelines describe that all patients with asthma should be provided with an asthma action plan to guide their self-management because it helps patients recognize and appropriately respond to the worsening of their asthma [6]. Although all patients receive such an action plan on paper during HACT, it is unknown to the HCPs if patients really use it. Although patients mentioned that written action plans (on paper) are often neglected, not received, or thrown in the trash, 88% (46/52) of them wanted the action plans to be available on their smartphone. By making it more accessible in an app, the action plan can be expanded and customized for the individual patient. Our suggestion is that the action plan can be the root of an app, whereas different additional functions mentioned as useful can be the possible branches. Although action plans are not often used in practice, they can help patients guide their self-management [6]. By adding an option to disable branches that are seen as irrelevant by or for a patient, we can prevent the patient from being overwhelmed. Personalization options are particularly important in this diverse group of patients with asthma, as indicated by the wide variety of potential goals and useful functions mentioned by patients and HCPs.

### Strengths and Limitations

This study has several strengths and limitations. A strength of this study is the specific patient population studied. Patients with asthma undergoing HACT are among the patients with most severe asthma in the Netherlands. As conventional therapy is unsuccessful in accomplishing sufficient levels of asthma control, these patients must consider other forms of asthma management. Patients undergoing HACT spend 3 months in the desolated Swiss mountains, away from their friends and family, because HACT was their only solution left to improve the quality of life. Any tool able to assist them in increasing their asthma control at home would, therefore, be appreciated, and they are more likely to adhere to an (future) mHealth system.

The mixed methods study design allows for quantifying the attitudes expressed in qualitative stage 1 of the study. In the interviews and focus groups, the HCPs and patients were able to fully express their thoughts and ideas about mHealth in 30-minute or 2-hour sessions. Full train of thoughts could be formed and discussed, allowing for thoughtful considerations as to what is important and useful. Using the questionnaire, we tested whether these opinions were more widely supported by other patients.

A limitation of this study is the relatively modest number of respondents to the questionnaire. Every year, until 2019, approximately 80 patients in the Netherlands were admitted to HACT, and many of these patients went more often than once. This resulted in a small study population, and further research could help us better understand this population. The respondents agreed with most of the attitudes expressed in the focus groups, and the inclusion of the questionnaire was a useful addition to this study. As the first part of the study was qualitative, there could always be a selection bias. There were a limited number of patients and HCPs in the focus groups and interviews. This could have influenced the results. As we went to Davos at a random moment in time and invited all the patients present at that time to participate, we expected to have tackled the potential selection bias in these focus groups. We also tried to counteract bias by sending out the questionnaire to a larger group in stage 2.

Many different functionalities for the mHealth system were named. In our study, we did not ask patients to order the importance of different options. Therefore, we do not know whether every function is of equal importance or whether some can be left out without affecting user satisfaction. In this study, we gathered information on what was important to the end users but not on how important these options were to them. This could be explored further in future studies.

### Comparison With Prior Work

In a previous study performed by Simpson et al [22], the perspectives of patients with general asthma and HCPs on mHealth were explored. Although most of the goals were similar to those of this study, the patients from the Simpson study reported that they wanted a system to assist them in emergency situations. The patients in this study did not mention this option. This could be attributed to the higher asthma severity of the patients in our study because they are more used to having severe asthma symptoms.

In a 2017 review of the available asthma apps, 38 different apps were analyzed [23]. A total of 42 functions in 4 categories were identified: tracking, information, assessment, and notifications. Most of the tracking (monitoring) functions mentioned in the focus groups were available in these apps, except for the tracking of specific health data (eg, heart rate and oxygen saturation) and lifestyle parameters (eg, weight and exercise). An additional information (education) option mentioned in the focus groups was information for friends and family. “Assessment” provides the interpretation of recorded values, including the availability of the asthma action plan. Finally, all notification options implemented in these apps were reported in the focus groups, including medication reminders.

Van der Kleij et al [14] reported on 6 conditions that they regarded as vital for the development and implementation of safe eHealth apps in primary care [14]. The first condition they named was “together: stakeholder engagement and co-creation.” An eHealth system must be improved in an iterative setting based on the attitudes of relevant stakeholders [24]. The second condition is called “blended: combining eHealth with regular care” in which it is outlined that an eHealth system is combined with regular face-to-face care. The third condition, “individualized and inclusive,” poses that personalizing eHealth has the potential to be more effective than a one-size-fits-all app [25]. The next condition is “ethical: being attentive of ethical considerations, privacy, and patient safety.” It is said, for example, that the privacy of patient data needs to be accounted for. These 4 conditions were named in our focus groups and interviews as the major topics. The condition “evidence-based: continuous research and educational guidance” will be met in the next phase of the Davos@home study when the effectiveness of the future mHealth system will be evaluated. The last condition “global: eHealth in primary care in high- and low-resource settings” is at the moment not applicable because Davos@home is entailing a specific patient population.

### Future Perspective

In the next Davos@home project, an mHealth system will be built based on the results of this study. The effectiveness of the system will be evaluated by assessing its effect on asthma control after the discharge of patients from HACT compared with the regular aftercare process. The use of mHealth to support the self-management of asthma opens up a lot of possibilities. Many different aims can be targeted when developing an mHealth system, which can be a pitfall. Therefore, it must be clear to the developers of the system what the goals of the end users are to enable cocreation. Although it might be challenging to create a system with different functionalities, it is better to invest in a system that meets most of its users’ demands than in a system that is unlikely to be used.

### Conclusions

Different patients reported that they would benefit the most from different functionalities. Therefore, it is important to tailor functionalities to individual (treatment) goals. When developing an mHealth intervention, it is important to allow personalization to avoid overwhelming the users.

## Abbreviations

ACQ: Asthma Control Questionnaire
HACT: high-altitude climate therapy
HCP: health care provider
mHealth: mobile health
